# Macaw Palm Propagation Strategies: Advances, Gaps, and Future Directions for a Promising Oleaginous Crop—A Review

**DOI:** 10.3390/plants15030488

**Published:** 2026-02-05

**Authors:** Vytória Piscitelli Cavalcanti, Laís da Silva Braga, Anna Carolina Abreu Francisco da Costa, José Victor Maurício de Jesus, Jorge Braga Ribeiro Junior, Heloisa Oliveira dos Santos, Rafael Peron Castro, Adão Felipe dos Santos, Joyce Dória

**Affiliations:** 1 Goiano Federal Institute of Education, Science and Technology, Rio Verde Campus, Rio Verde 75901-970, GO, Brazil; vytoriapc27@gmail.com; 2 Department of Agriculture, Federal University of Lavras (UFLA), P.O. Box 3037, Lavras 37200-900, MG, Brazil; lais.braga@estudante.ufla.br (L.d.S.B.); anna.silva15@estudante.ufla.br (A.C.A.F.d.C.); jose.jesus2@estudante.ufla.br (J.V.M.d.J.); jorge.junior1@estudante.ufla.br (J.B.R.J.); heloisa.osantos@ufla.br (H.O.d.S.); rafael.peron@ufla.br (R.P.C.); adao.felipe@ufla.br (A.F.d.S.)

**Keywords:** *Acrocomia aculeata*, sexual propagation, asexual propagation, micropropagation, cutting propagation

## Abstract

The *Acrocomia aculeata* is a promising palm tree for biofuel production, but it faces challenges related to propagation, especially due to seed dormancy. This article presents an integrative review, supported by bibliometrics, of the sexual and asexual propagation methods of the species, conducted through searches in Scopus, SciELO, and Web of Science databases. The results indicate that sexual propagation is the predominant approach in the literature, although it faces significant challenges due to seed dormancy, such as the physical resistance to embryo protrusion imposed by the operculum. Asexual propagation demonstrates great potential through micropropagation techniques, which allow obtaining genetically uniform plants in relatively short periods. The non-deep physic dormancy exhibited by the seeds interferes with germination by constraining embryo growth potential and postponing the metabolic reactivation essential for successful germination. Despite the existence of promising methods for overcoming dormancy, additional studies are needed to understand the mechanisms involved in this process. This review maps the scientific literature to highlight areas of proven research success, identify critical gaps and underexplored topics, and indicate how future investigations can support the development of efficient propagation protocols and the establishment of commercial plantations.

## 1. Introduction

### 1.1. Economic, Environmental, and Industrial Relevance of Macaw Palm

The macaw palm (*Acrocomia aculeata* (Jacq.) Lodd. ex Mart.) has attracted significant and continually growing interest owing to its substantial economic and environmental potential. Reflecting current industrial utilization trends, it is increasingly recognized for its diverse applications across cosmetics, food, and energy sectors [1,2,3,4,5]. Its particular prominence in biofuel production is well documented by both foundational [6,7,8] and very recent, forward-looking studies [9]. This broad and sustained interest is primarily driven by the high content and quality of the oil extracted from its fruits and the use of its by-products, such as biomass [2,3,4,5,10] and its crucial role in carbon accumulation for climate change mitigation [11]. Macaw palm may sequester up to approximately 350% more carbon than African oil palm, according to estimates of greenhouse gas emission balances [12].

The increasing demand for renewable energy sources and global imperatives for sustainable agriculture strongly incentivize the replacement of petroleum with biofuels. In this context, the macaw palm is a prominent candidate, distinguished by its high oil productivity—comparable to that of oil palm (*Elaeis guineensis*)—and superior biofuel quality [13,14,15].

### 1.2. Distribution, Adaptability, and Contribution to Sustainability Goals

The species’ remarkable edaphoclimatic adaptability facilitates cultivation in degraded areas and seamless integration into agroforestry systems [16]. The species’ extensive distribution across Brazil and other South American countries [17,18,19], coupled with a supply chain that avoids competition with food production, solidifies its competitive edge in the biofuel industry [9].

The expansion of the macaw palm production chain, driven by its unique ability to thrive in degraded areas and integrate into agroforestry systems [16], positions its supply chain to largely avoid competition with conventional food production [9]. This characteristic is crucial, as it directly aligns with global environmental objectives, such as the United Nations Sustainable Development Goals (SDGs) and Nationally Determined Contributions (NDCs) under the Paris Agreement. By restoring degraded areas and promoting sustainable farming practices (SDGs 12 and 15) without displacing food crops, *A. aculeata* contributes to clean energy (SDG 7), creates new jobs and supports economic development (SDG 8), and significantly aids in mitigating climate change by reducing greenhouse gas emissions (SDG 13), a benefit further underscored by dedicated quantitative research on its carbon accumulation capacity [11,20].

### 1.3. Biological Traits and Challenges for Domestication

The broad distribution of macaw palm may be associated with the species’ hardiness, as it exhibits great adaptability to diverse edaphoclimatic conditions, growing across a wide range of soil types and tolerating significant climatic variations [21]. One factor that may contribute to this adaptability is allogamy, a reproductive strategy that promotes genetic variability and can influence the species’ resilience to adverse environmental conditions [22].

However, despite its great commercial potential, the domestication of the macaw palm still faces challenges that limit its large-scale production [21]. The acquisition of viable seeds is particularly hampered by the inherent complexity of fruit harvesting and processing. This complexity stems from the fruit’s rigid anatomy, including its tough exocarp and lignified endocarp, which makes mechanical dehusking challenging and labor-intensive, thus constraining efficient seed separation; however, these same structural features contribute to protecting the kernel, reducing oxidative degradation, and ultimately preserving the quality of the extracted oil [23]. Additionally, seed dormancy represents a significant obstacle to the species’ propagation, as many viable seeds fail to germinate under favorable environmental conditions, resulting in a slow and irregular germination process [24,25,26,27]. Such characteristics collectively compromise commercial production, as the low germination rate and speed make large-scale seedling production arduous, particularly because the macaw palm does not exhibit natural vegetative propagation, making seed-based propagation the primary option for plantation establishment [28,29,30].

### 1.4. Advances in Propagation Techniques and the Role of Biotechnology

Several methods have been tested to overcome dormancy and improve the germination rate of macaw palm, including the use of plant growth regulators, operculum removal, and controlled environments [31,32,33]. While these approaches demonstrate varying degrees of efficacy, their comparative scalability and economic feasibility under commercial nursery conditions are complex factors that warrant further dedicated investigation; however, our review herein aims to analyze existing methods for breaking macaw palm seed dormancy and for in vitro micropropagation, and to identify the most promising approaches in terms of biological effectiveness.

Given these challenges and advancements, biotechnology emerges as a key strategy to support the initial steps toward large-scale cultivation and the establishment of commercial macaw palm plantations. Tissue culture techniques, for instance, have been explored as an alternative to overcome slow germination challenges and to obtain obtaining plants from somatic embryos that are genetically uniform and produced in a shorter time [34,35,36]. Such approaches are particularly relevant for perennial palms with long juvenile phases and wide genetic variability, as they enable the large-scale clonal propagation of elite genotypes with desirable agronomic traits. In other palm species, such as oil palm and coconut, micropropagation has already proven valuable for commercial-scale multiplication [37,38], reinforcing its potential applicability to macaw palm. However, for *A. aculeata*, studies remain fragmented, with challenges related to the selection of explants, culture media, and growth regulators, as well as the need to overcome limitations in embryogenic competence. In parallel, the development and optimization of methods for breaking seed dormancy remain critical for sexual propagation. Together, these limitations delineate a clear research frontier, in which advances in both asexual propagation and seed dormancy-breaking protocols could decisively contribute to the conservation of *A. aculeata* and its commercial cultivation.

Thus, the aim was to conduct an integrative review of sexual and asexual propagation methods for *A. aculeata*, integrating and systematizing the main methodologies reported in the literature to summarize key findings, highlight underexplored research areas, and guide future studies focused on overcoming propagation constraints and supporting the commercial expansion of this palm species.

## 2. Results and Discussion

The bibliometric analysis was conducted on a dataset comprising 35 documents published between 2008 and 2024, indexed across 25 different sources, including journals and other publication outlets. The temporal distribution of publications indicates a relatively recent and still developing research field, with an annual growth rate of 4.43%, suggesting a gradual expansion of scientific interest over time.

The corpus comprised 104 authors and 166 appearances, indicating a relatively broad authorship base. No single-authored documents were identified, highlighting a strong tendency toward collaborative research in the field. On average, each document included 4.74 co-authors, while the number of documents per author was 0.337, reflecting a dispersed authorship structure with limited recurrence of the same authors across publications.

**Figure 1:**
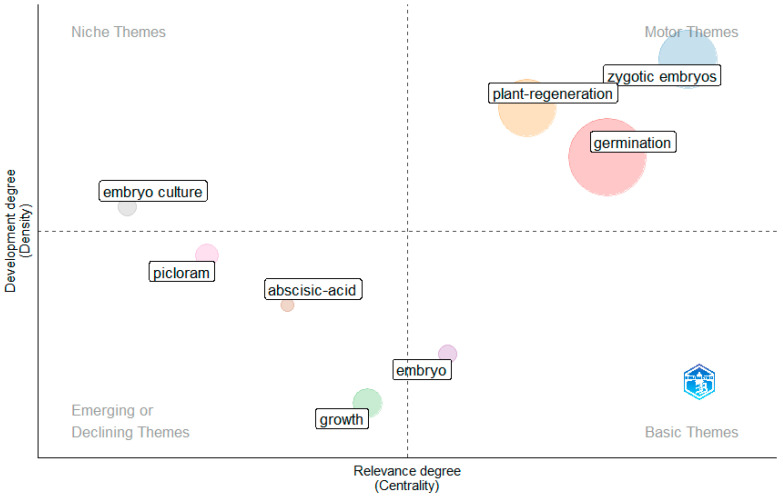
Thematic map of research on the propagation of *A. aculeata*.

The thematic map revealed a research field strongly structured around germination, plant regeneration, and zygotic embryos, which were classified as motor themes due to their high centrality and density (Figure 1). These themes represent the conceptual and methodological core of studies on macaw palm propagation. The theme embryo emerged as a basic theme, indicating its fundamental role across studies, albeit with lower internal thematic development. In contrast, embryo culture appeared as a niche theme, reflecting a specialized but less integrated research line. Themes related to growth and plant growth regulators, such as abscisic acid and picloram, were identified as emerging or declining, suggesting limited consolidation or reduced emphasis in recent literature.

Separate keyword co-occurrence networks were constructed for studies addressing the sexual and asexual propagation strategies of *A. aculeata* to explore thematic differences between approaches (Figure S1).

In the sexual propagation network, germination emerged as the most central and frequent term, showing strong co-occurrence with dormancy, zygotic embryos, endosperm, and embryos. This configuration highlights the predominance of studies focused on dormancy-breaking mechanisms, seed physiology, and early developmental processes. Peripheral terms such as growth, histochemistry, and oil indicate complementary analyses related to physiological performance and biochemical composition during germination.

In contrast, the asexual propagation network was structured around somatic embryogenesis, somatic embryo, and plant regeneration, reflecting the technical core of tissue culture–based approaches. The frequent association with zygotic embryos, embryonic development, and histology suggests that many studies rely on embryonic explants and emphasize detailed morphological and anatomical characterization of regeneration processes. Less frequent terms, such as leaf and monocotyledon, indicate alternative explant sources that were explored on a more limited scale.

The networks reveal a clear thematic distinction between propagation strategies: sexual propagation studies emphasize physiological and developmental aspects of germination, while asexual propagation research is more specialized and methodologically oriented toward somatic embryogenesis and in vitro regeneration.

**Figure 2:**
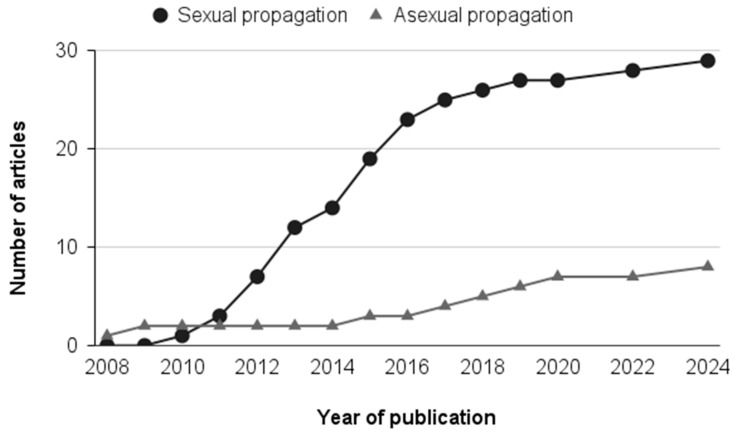
Cumulative number of articles published over time on sexual and asexual propagation of *A. aculeata*.

Among the 35 selected articles, 77.14% investigated only sexual propagation, 17.14% only asexual propagation, and 5.71% investigated both types of propagation. The temporal distribution of publications reveals distinct trends in research focused on sexual and asexual propagation strategies of *A. aculeata* (Figure 2). While the first publication on asexual propagation of *A. aculeata* appeared in 2008, studies focusing on sexual propagation emerged slightly later, with the first recorded in 2010. However, despite this later start, sexual propagation has since received significantly more attention, with the majority of studies appearing post-2010, indicating it has been the most studied method over time. Research on sexual propagation exhibited a marked increase between 2011 and 2016, reaching a peak around 2013–2015, when the highest annual number of publications was recorded. This period reflects intensified interest in seed germination, overcoming dormancy, and early seedling development. In contrast, studies addressing asexual propagation remained scarce and discontinuous over time. Publications related to in vitro techniques, somatic embryogenesis, and vegetative propagation appeared sporadically, with most years reporting either no publications or a single study. This pattern suggests that asexual propagation of macaw palm remains technically challenging and has not yet reached the same level of methodological consolidation as sexual propagation.

This trend highlights a strategic emphasis in research: even with the early exploration of asexual methods, the scientific community subsequently prioritized understanding and optimizing sexual propagation to address foundational challenges like seed dormancy. This focus on sexual reproduction post-2010 aligns strongly with both conservation and commercial cultivation priorities. For conservation, it is critical for maintaining genetic diversity and adaptability, while for commercialization, overcoming dormancy in seeds [19,31,32,39,40,41,42,43,44,45,46] is essential for developing robust breeding programs and providing the initial, genetically diverse plant material necessary for domestication and subsequent large-scale production, even when asexual methods are later employed for clonal propagation of elite lines.

**Figure 3:**
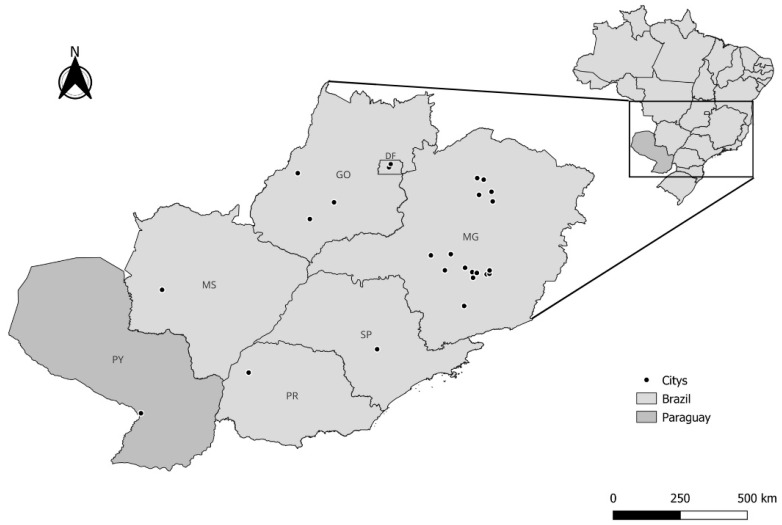
Map with collection locations of *A. aculeata* fruits reported in the analyzed articles. Brazil—MG: Santa Luzia, Itaúna, Pará de Minas, Montes Claros, Mirabela, Brasília de Minas, Bocaiúva, Belo Horizonte, Pitangui, Abaeté, Luz, Contagem, Florestal, Rio Paranaíba, Ijaci, São João da Lagoa, PR: Umuarama; GO: Montes Claros de Goiás, Rio Verde, Indiara; MS: Bodoquena; DF: Brasília, Sobradinho. Paraguay: San Lorenzo.

In the studies analyzed in this integrative review, the fruits of *A. aculeata* were predominantly obtained in Brazil and Paraguay (Figure 3). A significant majority of the samples were collected from the Center-South region of Brazil, especially the state of Minas Gerais, where mother plants and/or natural populations were identified in 16 cities, with the North of Minas region concentrating 54.3% of the total collections, and Montes Claros alone accounting for 40%. This geographical concentration in the analyzed literature, while reflecting current research trends, suggests a potential regional bias that could impact the generalizability of propagation findings, particularly concerning genetic diversity and localized adaptation across the species’ broader range.

Analyzing the distribution of publications by institution in research on *A. aculeata* propagation, a high contribution from Brazilian universities and research centers was observed (Figure S2). The pronounced geographical concentration of propagation and genetic studies on *A. aculeata* in Minas Gerais, Brazil, also reflects the historical consolidation of germplasm resources, research infrastructure, and production-oriented initiatives in this region. A key driver was the establishment of the BAG–Macaúba germplasm bank by the Federal University of Viçosa in 2009, one of the largest ex situ collections of *A. aculeata* in South America, comprising accessions derived from natural populations across nearly all Brazilian regions [12]. This collection, together with additional experimental plantations and adult palm collections maintained by research institutions such as the Agronomic Institute of Campinas, has provided accessible and well-characterized plant material for studies on germination, propagation, genetic diversity, and productivity [47]. Moreover, Minas Gerais has served as a primary testing ground for silvopastoral and agrisilvicultural systems involving *Acrocomia* spp., supported by regional development programs and public–private initiatives aimed at bioenergy and sustainable land-use systems [48,49]. However, despite the species-wide natural distribution across Latin America, comparable research efforts, germplasm collections, and long-term experimental platforms are lacking in other countries and regions. This geographical imbalance is consistent with the fact that *A. aculeata* research is still in an early development phase, with recognized gaps in genotype–environment interactions, planting material improvement, and sustainable cultivation systems. Consequently, the current literature, heavily centered on a restricted geographic context, may limit the generalization of findings across genetically and ecologically distinct populations, underscoring the need for expanded multi-regional studies and sampling.

Indeed, *A. aculeata* is known to occur across a wider territory, encompassing approximately half of Minas Gerais [50] and extending into other South American countries such as Argentina, Bolivia, and Paraguay [51,52,53]. However, our integrative review did not identify specific propagation studies originating from these broader regions that met our defined search and inclusion criteria for detailed comparative analysis. This highlights a clear research gap and an opportunity for future studies to investigate the propagation behavior of *A. aculeata* across its entire natural distribution.

### 2.1. Sexual Propagation

Sexual propagation is the main way in which palm species multiply. Although some can be propagated vegetatively, most of them in nature multiply predominantly by seeds [18]. This pattern is due to the absence of natural vegetative propagation mechanisms in most palms, which have only one apical meristem. As a result, their growth is restricted to the apex, preventing the formation of lateral shoots, as occurs in other plants [54,55,56].

Even though seed propagation is the main mechanism for multiplying palm trees, its efficiency is often limited by germination challenges, which are usually slow and irregular [39,57]. The complexity of this process stems from various dormancy mechanisms and the particular morpho-anatomical characteristics of the species, which influence the rate and uniformity of germination [41,57]. In this context, it is evident that dormancy in palm seeds is modulated mainly by their structural components [19].

**Figure 4:**
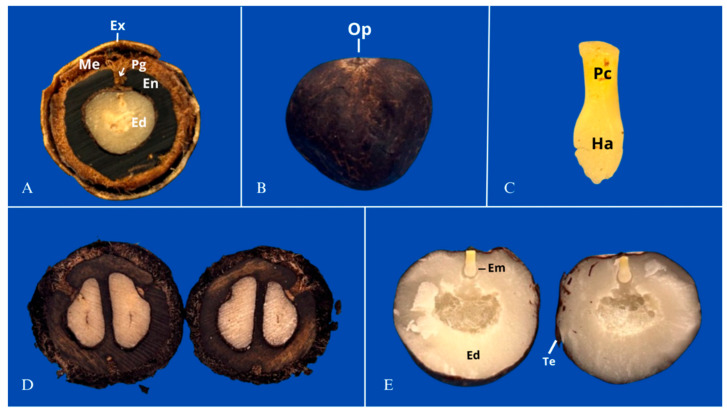
(**A**) Transverse section of a dried fruit of *A. aculeata* collected after storage. It is possible to verify the presence of the exocarp (Ex), mesocarp (Me), the germination pore (Pg) filled with mesocarp tissue (arrow), endocarp (En), and endosperm (Ed); (**B**) Intact seed of *A. aculeata*, with indication of the location of the operculum (Op). (**C**) Embryo, with indication of the cotyledonary petiole (Pc) and haustorium (Ha). (**D**) Transverse section of a dried fruit of *A. aculeata* containing two seeds. (**E**) Transverse section of a seed with indication of the location of the embryo (Em), endosperm (Ed), and the tegument (Te).

There is abundant evidence regarding the challenges of propagating *A. aculeata* by seed. Ribeiro et al. [39] reported that the germination of *A*. *aculeata* seeds under natural conditions is remarkably low, reaching as little as 3%, and can be prolonged for several years. Similarly, Rodrigues Junior et al. [44] observed low germination percentages (less than 5%) in seeds not subjected to any pre-germination treatment. From an ecological standpoint, this strong and prolonged dormancy, while hindering commercial propagation, is understood as a crucial adaptive trait for survival in the wild. It enables the species to form persistent soil seed banks, spreading the risk of germination over time and ensuring survival through fluctuating environmental conditions—such as unpredictable rainfall or seasonal fires—by only germinating when conditions are highly favorable for seedling establishment [45]. Research reveals that the pronounced dormancy is related to the low growth potential of the embryo, which is unable to overcome the mechanical restriction imposed by the operculum [19,32,39,41]. Figure 4 illustrates the main morphological components of fruits and seeds of *A. aculeata*.

In this context, it is noteworthy that several types of dormancies previously suggested for the species have been ruled out. Rodrigues Junior et al. [44] found that the species lacks physical dormancy, as the integument is very thin and provides no significant barrier to water absorption. Ribeiro et al. [58] confirmed the absence of morphological dormancy in the species, as they found that the embryo’s intermediate degree of differentiation does not restrict germination, and Ribeiro et al. [32] demonstrated that the integument and endosperm tissues do not chemically inhibit germination or initial seedling development.

Most of the studies analyzed report that the dormancy present in macaw palm seeds is of the non-deep physiological type, in which the embryo has low growth potential and is unable to overcome the restriction imposed by the operculum, a structure formed by the opercular integument and micropylar endosperm [19,40]. In this context, the operculum does not limit water absorption but is exclusively associated with mechanical restrictions on embryo growth. During germination, the growth of the embryo promotes the displacement of the operculum and the protrusion of the cotyledonary petiole, which is considered indicative of germination in the species [41].

**Figure 5:**
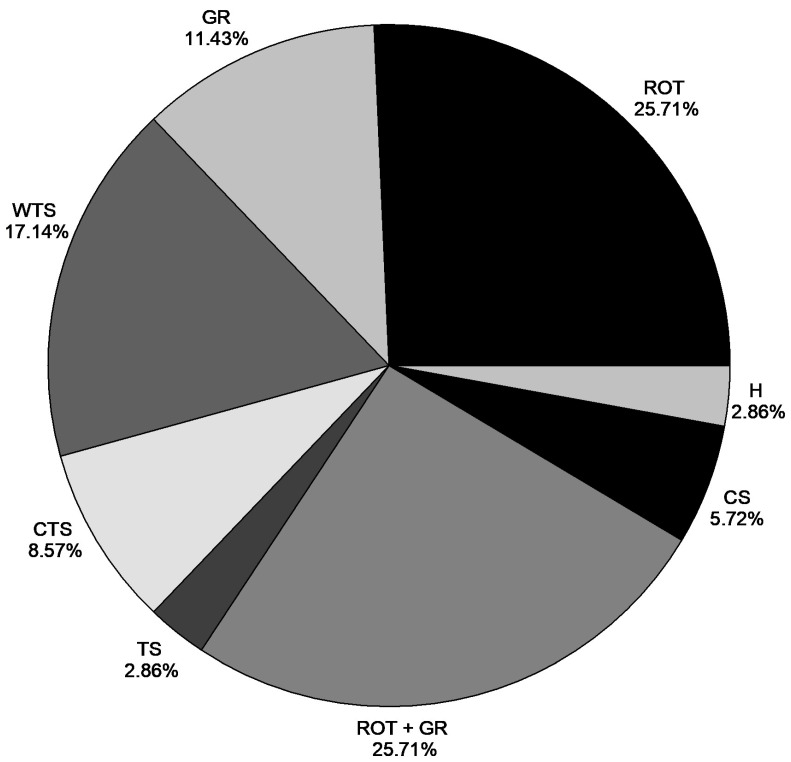
Methods for overcoming dormancy in *A. aculeata* seeds reported in the literature. The results are expressed as the percentage of research testing each method for overcoming dormancy. Removal of the opercular tegument (ROT); Growth regulators (GR); Warm thermal stratification (WTS); Cold thermal stratification (CTS); Thermal stratification (combination of cold and warm) (TS); Removal of the opercular tegument + Growth regulator (ROT + GR); Chemical scarification (CS) and Hydration (H).

Most studies report the need for pre-germination treatments to increase the germination rate and uniformity of *A*. *aculeata* seeds. Figure 5 shows the different methods used to overcome macaw palm seed dormancy, as reported in the articles in which the species was propagated ex vitro. Among the pre-germination treatments, the removal of the opercular integument and the systematic combination of operculum removal with the use of growth regulators were the main methods used to promote seed dormancy breaking, both occurring in 25.71% of studies. While these mechanical and hormonal combinations have been explored, a deeper systematic investigation into more complex, multi-factorial treatments—such as integrating thermal stratification with chemical scarification and hormonal applications—remains a research gap worth addressing. On the other hand, warm thermal stratification and the sole use of growth regulators represent 17.14% and 11.43%, respectively, of the studies evaluated. Other pre-germination treatments for dormancy breaking, such as cold thermal stratification (8.57%), chemical scarification (5.72%), exposure of seeds to cold and heat (2.86%), and seed hydration (2.86%), have also been reported in the literature.

After operculum removal, Ribeiro et al. [40] observed the occurrence of protrusion of the cotyledon petiole at seven days after sowing, resulting in a germination rate of 86% at 15 days. However, in seeds kept with the operculum, there was no germination. The authors argue that the removal of the operculum may favor the growth of the embryo due to greater availability of oxygen and greater exposure of the embryo to the atmosphere. In addition, the removal of the opercular integument increases the speed of water absorption by the embryo [43].

Carvalho et al. [41] also showed that no germination occurred in intact seeds; however, 96 h after removal of the opercular integument, a final germination rate of 60% was obtained. Compared to other palm species, the authors reported that *A. aculeata* has more pronounced dormancy, a fact that is directly linked to the morphoanatomy of the opercular lining. For the seeds of this palm tree, the operculum is twice as thick as that of other species, being composed of more juxtaposed cells without a longitudinal alignment of the cell walls, thus making it difficult to rupture them [41].

Ribeiro et al. [39] evaluated the efficacy of the combination of operculum removal and the use of growth regulators through the application of gibberellin (GA_3_) at different concentrations in seeds with and without operculum. The application of GA_3_ resulted in germination percentages higher than those observed in intact seeds. The removal of the operculum combined with a concentration of 2000 mg L^−1^ of GA_3_ was the most efficient treatment, resulting in 50% germination. The positive effects of this combination on germination were also found by Oliveira et al. [59], Rodrigues Junior et al. [43] and Mazzottini-dos-Santos et al. [19].

Although the percentage of studies that used hot thermal stratification to overcome dormancy in macaw palm seeds is high, its efficacy in promoting germination has not been proven. In the study carried out by Rubio Neto et al. [60], it was found that the results of hot stratification did not differ from those obtained in the control treatment and presented germination percentages and germination speed lower than those obtained with the removal of the opercular integument. Similarly, Rodrigues Junior et al. [44] found that exposure of seeds to high temperatures did not promote increased germination and also contributed to a loss of viability over time, which may be associated with the thin integument and the absence of physical dormancy in macaw palm seeds.

However, in nature, the occurrence of high temperatures, such as those caused by fire, is important for the breakdown of physiological dormancy of some species, by reducing the endogenous levels of abscisic acid (ABA). For macaw palm seeds, the effects of thermal stratification on overcoming dormancy will need to be studied in greater detail; however, the seeds demonstrate considerable tolerance to prolonged conditions of high temperatures, representing an adaptive strategy in their natural habitat [44].

The use of growth regulators has been an important strategy for the breakdown of physiological dormancy in different species. Gibberellins, for example, stimulate germination by inducing the synthesis and/or activation of enzymes that loosen the cell wall, acting as a stimulus for the protrusion of the embryo/radicle and allowing the completion of germination [61]. In *A. aculeata*, studies indicate that gibberellins act to weaken the tissues adjacent to the operculum, allowing the displacement of this structure and, consequently, promoting embryo growth [32,41].

Bicalho et al. [42] reported that the pre-germination treatment of seeds with GA_3_ resulted in a four-fold increase in germination percentage and was accompanied by a reduction in germination time compared to seeds without GA_3_. In addition, the authors found that seeds with GA_3_ reached maximum germination at four weeks, while seeds without GA_3_ reached maximum germination potential at 18 weeks. Ribeiro et al. [39] and Oliveira et al. [59] found that the exposure of seeds to high concentrations of GA_3_ promoted a 30 to 50% increase in the germination rate of macaw palm seeds.

Although GA_3_ promotes significant effects in the promotion of germination in palm seeds, for *A. aculeata* lower percentages have been observed when compared to the removal of the operculum. Bicalho et al. [42] explained that the low germination of macaw palm seeds treated with GA_3_ suggests different depths of dormancy and, as a consequence, different sensitivities to GA_3_ within the seed population. It is noteworthy that the removal of the operculum induces germination in the short term, while the application of GA_3_ favors the germination of this palm in the long term [40]. This difference is due to the fact that GA_3_ requires a longer period to interact with the tissues adjacent to the embryo and induce its weakening, while the removal of the operculum immediately eliminates the physical barrier to the emergence of the germinal bud, resulting in a faster germination response.

**Figure 6:**
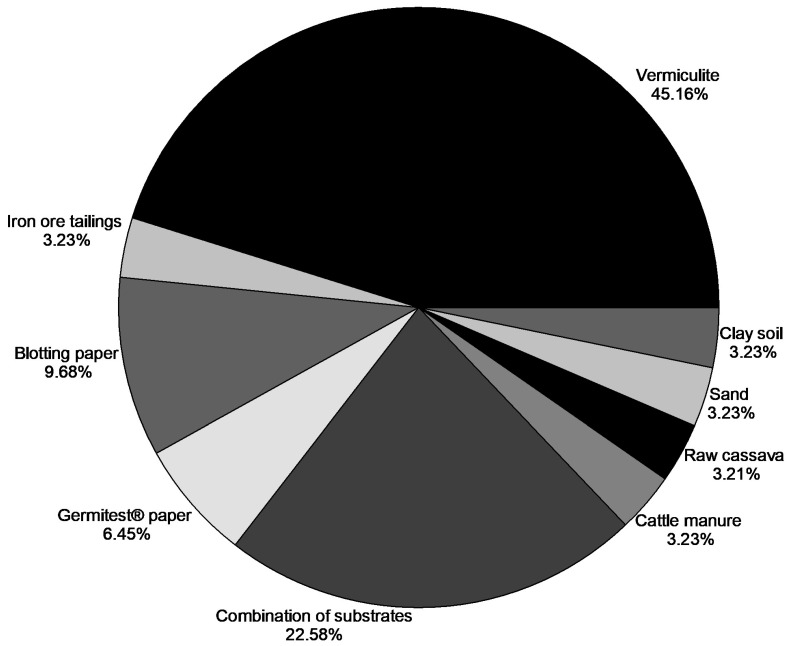
Types of substrates most commonly used in the ex vitro sexual propagation of *A. aculeata* seeds, as reported in the literature. The results are expressed as the percentage of research testing each type of substrate.

As for the types of substrate used in studies involving ex vitro sexual propagation (Figure 6), vermiculite was the most common, being used in approximately 45.16% of the studies. Combinations of substrates were the second most used option, accounting for around 22.58%. Vermiculite is a type of substrate widely used in plant propagation, and in Brazil, its use has been recommended by official institutions. *Ministério da Agricultura e Pecuária* (MAPA) Instructions for Seed Analysis of Forest Species recommends the use of vermiculite as a substrate for germinating *A. aculeata* seeds. It is important to highlight that vermiculite is a mineral similar to mica, composed mainly of hydrated silicates of aluminum and magnesium [41]. It is a substrate commonly used for seed germination and is highly recommended for use in seed analysis laboratories as a substrate for the standard germination test, due to its numerous advantages such as low density, uniformity in chemical and granulometric composition, porosity, water retention capacity, and it is also considered a sterile product [41,62].

Among the substrate combinations reported in the studies, for example, the use of soil and sand in a 2:1 ratio [46], soil, sand, and humus in a 2:1:1 ratio [63], and 50% cattle manure and 50% vermiculite [64] were found. However, our review reveals that the specific physiological requirements of *A. aculeata* guiding the selection of these formulations, as well as a systematic evaluation of critical substrate properties such as pH, water retention capacity, and nutrient composition, were largely underexplored across these studies. Although these formulations have been tested, there are still few studies that systematically evaluate the influence of substrates on the germination and establishment of macaw palm seedlings, showing that basic studies are still needed for propagation to align with species-specific needs and indeed represents a significant research gap for optimizing propagation protocols.

Among the few studies available, Costa et al. [64] found that substrates containing manure are indicated for the formation of macaw palm seedlings, indicating that the components present in this material can favor the initial development of the species. Corroborating the adaptability of macaw palm to different cultivation conditions, Alves et al. [16] observed that its seeds have a high germination rate and initial establishment capacity, even when grown in iron ore tailings. In this sense, the authors highlight that *A. aculeata* is a promising species to be used in the rehabilitation of areas impacted by the deposition of tailings, due to its physiological characteristics and great resistance.

The presence of microorganisms in the seeds and/or during the conduct of germination tests can compromise the safety of the results and the propagation process as a whole. In the analyzed studies, it was found that sodium hypochlorite (36.36%) and chlorine solution (15.15%) are the main agents used in the seed disinfection process. Concentrations of 2.5%, 5%, 6% and 10% of the commercial sodium hypochlorite solution were used [16,45,63,65]. The time of exposure of the seeds to the solution ranged from 5 to 30 min. While these protocols vary widely, our review found no systematic comparative studies examining seed viability or embryo damage directly resulting from these diverse treatments. Similarly, although “chlorine solution” was used less frequently, the reviewed literature does not provide evidence to suggest better efficacy, lower phytotoxicity, or other advantages that would differentiate its use from sodium hypochlorite. It is worth noting that sodium hypochlorite is typically found in higher concentrations, commonly between 10% and 13% active chlorine, whereas chlorine solutions generally contain between 1% and 5% active chlorine. This difference in available concentration might influence their application and perceived effectiveness.

Moreover, despite disinfecting the seeds, some authors argue that it is still necessary to treat the seeds, especially with fungicides. This need is due to its chemical composition, since due to the large amount of reserves present in macaw palm seeds, combined with the duration of germination evaluations, they provide favorable conditions for the development and proliferation of pathogens.

In addition to disinfecting the seeds with the sodium hypochlorite solution, Carvalho et al. [41] and Souza et al. [45], also treated seeds using the commercial fungicide Derosal Plus^®^, in order to avoid contamination by pathogens. Derosal Plus^®^ contains the active ingredients Carbendazim and Thiram. Carbendazim, a Benzimidazole fungicide, acts systemically by inhibiting fungal growth through interference with tubulin biosynthesis. Thiram, a Dimethyldithiocarbamate fungicide, provides both contact and systemic effects, also inhibiting tubulin biosynthesis. These compounds are widely used for seed treatment in various crops such as cotton, rice, beans, corn, and soybeans. It is worth noting that in Brazil, there are no registered molecules for the treatment of macaw palm seeds, and studies that identify potential molecules for this purpose are of great relevance, highlighting the need for careful evaluations related to potential phytotoxic effects and residual chemical effects on germination.

**Figure 7:**
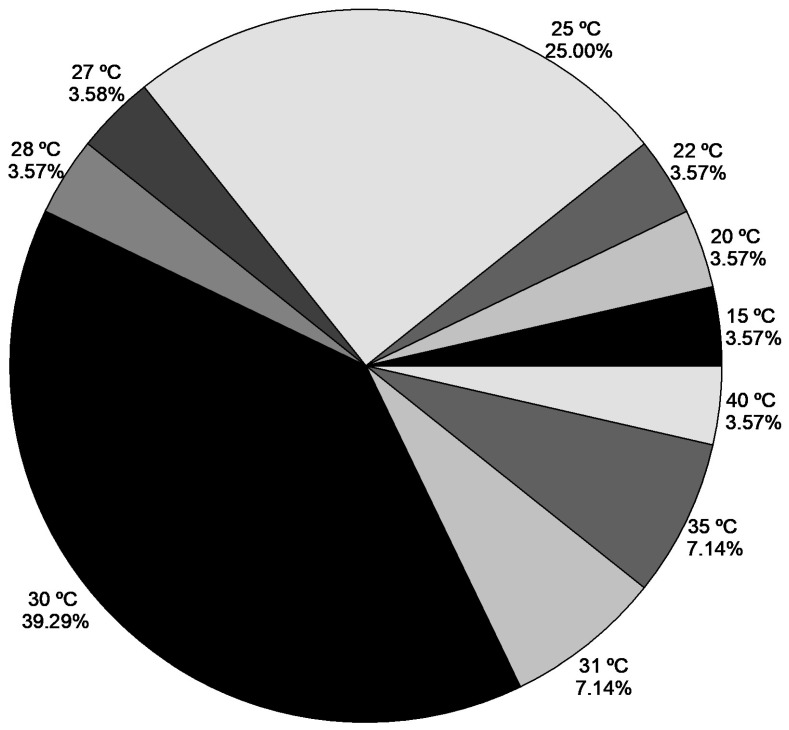
Temperature used in ex vitro sexual propagation in the studies evaluated. The results are expressed as the percentage of research testing each temperature.

The temperatures used in the evaluated studies ranged from 15 °C to 40 °C, with temperatures of 30 °C and 25 °C being used more frequently, with 39.29% and 25%, respectively (Figure 7). In the experiments conducted under laboratory conditions by Souza et al. [45], seeds with absence of the opercular seed coat showed the highest germination percentages at temperatures between 20 and 40 °C, while at 15 °C the lowest germination percentages were observed. These findings suggest that once the primary mechanical dormancy imposed by the operculum is overcome, temperature critically influences the embryo’s growth potential, directly impacting its ability to complete germination. The authors pointed out that in seeds with intact operculum there was no germination during the time they were kept at different temperatures emphasizing the operculum’s role as a primary barrier. However, after exposure to the aforementioned temperatures, the seeds were transferred to 30 °C, resulting in a considerable increase in germination for those without the operculum, indicating the temperature’s role in optimizing physiological processes for embryo development.

The results of Souza et al. [45] emphasize that high temperatures are important for breaking seed dormancy. Seedling growth potential tends to occur after periods of low temperature range, typical of winter, followed by rising temperatures in early spring, indicating the beginning of the rainy season in nature.

For plants in subtropical and tropical climates, the ideal temperature range for growth and dry-matter accumulation is 20–30 °C; lower or higher temperatures can compromise seedling development [66]. For macaw palm, low temperatures limit germination; however, periods with low temperatures can act as a stratification treatment and can favor germination when temperatures rise and reach the ideal range for the species [39].

The main articles on germination and overcoming dormancy in *A. aculeata* seeds were selected, highlighting their relevance and the key results. Table 1 summarizes these studies, allowing comparison of different methodological strategies and identification of conditions that yielded higher germination percentages and better maintenance of seed viability.

In vitro sexual propagation, performed through the culture of zygotic embryos of *A. aculeata*, was also evaluated in the selected articles. The main results reported are summarized as shown in Table 2.

**Figure 8:**
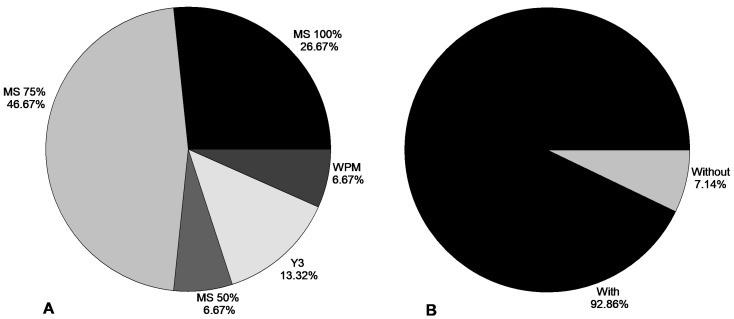
Culture media (**A**) and use of activated charcoal (**B**) in the sexual propagation of zygotic embryos in vitro. The results are expressed as the percentage of research testing each culture media (**A**) or testing the media with or without activated charcoal (**B**).

The most used culture medium was the medium described by Murashige and Skoog [68] (MS), in predominant concentrations of 75% and 100%, such concentrations were reported in the studies 46.67% and 26.67%, respectively (Figure 8A). The use of activated charcoal was observed in 92.86% of the studies evaluated (Figure 8B).

In in vitro culture, activated charcoal is often used to improve cell growth and development. In addition to contributing to the reduction in phenolic oxidation and exudates, it also favors the establishment of a dark environment in order to simulate soil conditions [69].

Different types and formulations of culture media, as well as the use of activated charcoal and growth regulators, are of great relevance for in vitro cultivation, especially for asexual plant propagation. Thus, in the studies analyzed in this research, the combination of different factors may induce different ways to obtain new plants, a subject that will be presented in the next topic.

Despite the large amount of research into sexual propagation, it is crucial to highlight that a major thrust of research has been directed towards establishing an efficient cloning protocol via somatic embryogenesis. This approach is highly sought after because, leveraging the principle of totipotency, any vegetative part of the plant possesses the inherent capacity to regenerate a complete plant genetically identical to the mother plant. Furthermore, from a single explant, numerous clones of the mother plant can be obtained, in stark contrast to a seed, which typically yields only one plant [67].

### 2.2. Asexual Propagation

Even though *A. aculeata* is a single-stemmed palm with a limited natural capacity for vegetative propagation, recent developments have demonstrated that asexual methods are feasible for this species. The technical potential for clonal multiplication is demonstrated by somatic embryogenesis protocols developed from zygotic embryos [70] and young leaf tissues [71]. This allows for the large-scale propagation of elite genotypes. This technique is particularly relevant to commercial cultivation because it allows for the rapid preservation of desired genetic traits, something that sexual reproduction alone cannot ensure. The species’ mixed reproductive system and high outcrossing rate also contribute to genetic variability and breeding programs [22]. Given the lengthy juvenile period and high genetic diversity of *A. aculeata*, asexual propagation techniques in this context supplement sexual propagation by offering resources for both conservation and commercialization.

Although asexual propagation has benefits, such as genetic uniformity, preservation of desirable characteristics, and shorter seedling production time, only 22.86% of the articles evaluated the asexual propagation of *A. aculeata*. It highlights the need for further studies on asexual propagation methods for *A. aculeata*. Cutting propagation (12.5% of the asexual propagation articles) and micropropagation through the production and germination/regeneration of somatic embryos in plant tissue culture (87.5%) were the methods reported.

**Figure 9:**
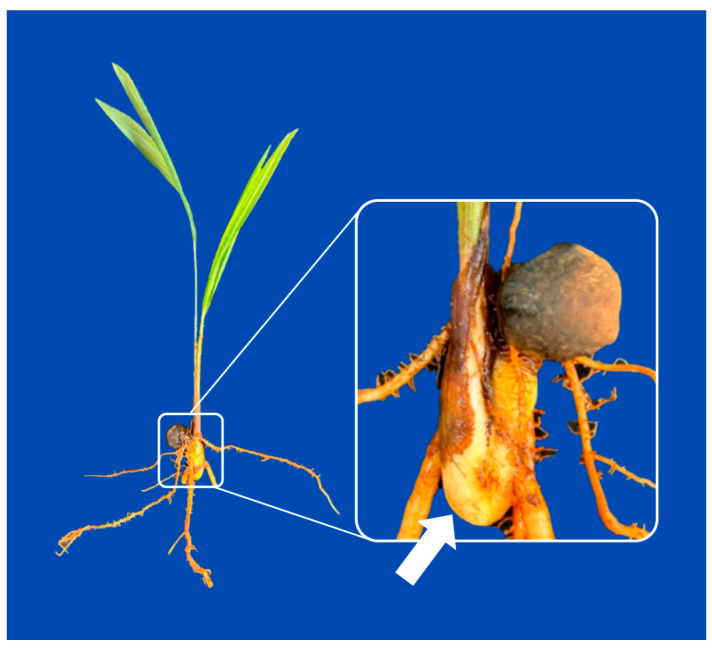
Plant of *A. aculeata* with a focus on the saxophone stem (white arrow).

Souza et al. [72] investigated the cutting propagation method for *A. aculeata* using excised saxophone stems (Figure 9) from 1- to 3-year-old plants, cultured for 60 days to evaluate root and shoot emission. The results showed that 52.5% of the samples showed growth in the aerial portion, while only 6% emitted roots. However, when the excised saxophone stems were stored for 90 days at room temperature, root emission increased to 30%. These findings suggest the need for further studies to optimize this method, such as using growth regulators to improve root emission and to evaluate the feasibility of this method for large-scale production.

The micropropagation method for *A. aculeata* consisted of callus induction, multiplication, and subsequent induction of somatic embryo formation, followed by somatic embryo maturation, germination, and plant regeneration, being a process that can be completed in less than 200 days [35]. The best results from each article for the micropropagation of *A. aculeata* are summarized in Table 1 and Table 2. In vitro culture and somatic embryogenesis have been the primary methods used to study the asexual propagation of *A. aculeata*, as they offer the possibility of large-scale multiplication of elite genotypes. However, several interrelated factors must be considered collectively rather than separately for these protocols to be successful. Explant disinfection is a crucial first step because contamination can hinder establishment in culture and jeopardize subsequent developmental responses. Therefore, the viability of tissues to react to culture media is directly impacted by the effectiveness of sterilization procedures.

Regarding the disinfection protocols used in the micropropagation of *A. aculeata*, sodium hypochlorite was identified as the main disinfecting agent for seeds, as well as for immature and unexpanded leaves, being used in all analyzed articles (Table 3). Before sodium hypochlorite soaking, some studies pre-treated seeds with 70% ethanol [70] or 0.01% mercuric chloride [73,74], while immature and unexpanded leaves were pre-treated with 70% ethanol [36,71]. In studies using zygotic embryos as explants, seeds were first disinfected with detergent. The exposure time of seeds to sodium hypochlorite ranged from 50 min to 12 h, whereas for immature and unexpanded leaves, it varied from 10 to 20 min. Sodium hypochlorite concentrations of 2.5% to 6% were used for seed disinfection, while concentrations between 1% and 2.5% were used for immature and unexpanded leaves (Table 3). The selection of the disinfecting agent, as well as its concentration and exposure time, is essential for minimizing contamination while ensuring the survival and successful in vitro establishment of the explants.

Once asepsis is achieved, the composition of the culture medium becomes decisive. Nutrient balance and supplementation provide the biochemical environment that interacts with the applied growth regulators. The selection and concentration of growth regulators in this context play a major role in regulating morphogenetic pathways such as callus induction, somatic embryo formation, and shoot or root regeneration. It should be emphasized that morphogenic competence is determined by the interaction between medium composition and hormonal balance, which must be optimized together.

The culture media used for *A. aculeata* micropropagation were Y3 [75] and MS [68]. Y3 medium was reported as the most used in all stages, from callus induction until somatic embryo germination/regeneration. In contrast, MS medium was used only in the somatic embryo induction and germination/regeneration stages (Table 3 and Table 4). Additionally, some articles used a modified Y3 medium incorporating Fe-EDTA and/or vitamins from the MS medium. However, since no articles were found comparing different culture media for the micropropagation of *A. aculeata*, it would be premature to suggest Y3 as definitively superior based solely on its prevalence. Furthermore, while the concept of complementing potential nutrient limitations in MS with targeted modifications is plausible, the reviewed literature does not explicitly report experimental validation of such modified MS media in a comparative context for *A. aculeata*. Thus, this gap can be filled by studying different culture media to find the best medium for each micropropagation stage.

The percentage of articles using a culture medium with activated charcoal progressively increased at each stage of the micropropagation process (Table 3 and Table 4). The charcoal addition in the medium helps to avoid tissue browning and can favor the somatic embryo formation and maturation [35,73,74]. While various concentrations were reported (e.g., 0.3 g L^−1^ to 3.0 g L^−1^), the reviewed literature does not explicitly detail the experimental methodologies or optimization processes undertaken to balance phenolic adsorption with nutrient and growth regulator availability for somatic embryo development. This indicates a gap in understanding how researchers specifically fine-tuned charcoal concentrations to mitigate potential negative effects on essential components while maximizing its beneficial role.

The formation of embryogenic callus of *A. aculeata*, as well as the germination/regeneration of the somatic embryos, can be affect by biotic factors, such as the genotype, age of the mother plant, type of the explant, and abiotic factors, such as light conditions, use of growth regulators [34,36,74].

**Figure 10:**
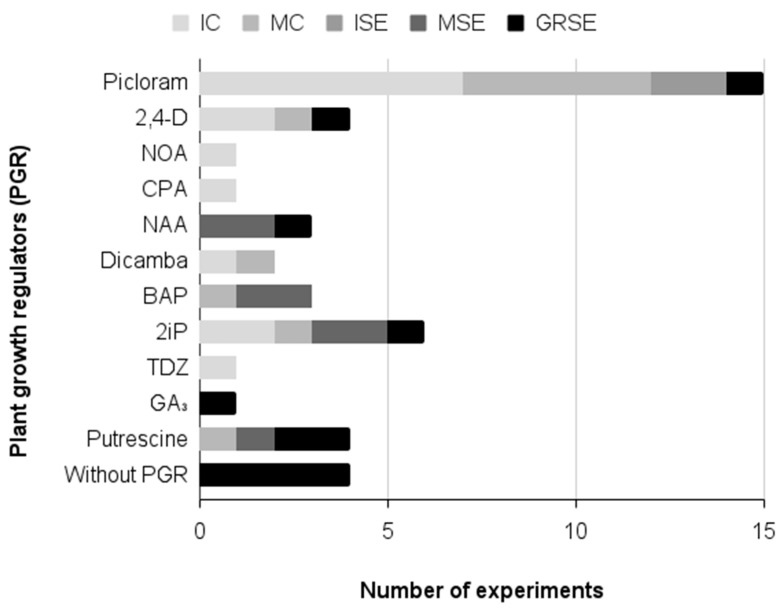
Plant growth regulators evaluated during *A. aculeata* micropropagation (IC = induction of callus, MC = multiplication of callus, ISE = induction of somatic embryos, MSE = multiplication of somatic embryos, and GRSE = germination/regeneration of somatic embryos). The results are expressed as the number of experiments testing each growth regulator, isolated or associated with another growth regulator, on each micropropagation stage. Picloram = 4-amino-3,5,6-trichloropicolonic acid; 2,4-D = 2,4-dichlorophenoxyacetic acid; NOA = Naphthoxy acetic acid; CPA = 4-chlorophenoxyacetic acid; NAA = Naphthaleneacetic acid; BAP = 6-Benzylaminopurine; 2iP = 2-isopenteniladenina; TDZ = Thidiazuron; GA_3_ = Gibberellic acid.

Figure 10 presents all of the plant growth regulators used in the experiments for each micropropagation stage of *A. aculeata*. Plant growth regulators were used in all experiments (100% of the analyzed articles) during callus induction and multiplication, as well as in somatic embryo induction and maturation (Figure 10). It is essential to use plant growth regulators in callus induction since callus formation does not occur in their absence [34]. Using auxins—mainly picloram and 2,4D—improved callus induction [35,36,71]. While for adding cytokinins—2iP, BAP and TDZ—no consensus was found in the literature, with some articles presenting no effect of adding cytokinins [36,73], and in another articles adding cytokinins improved the obtention of embryogenic lines and the maturation of somatic embryos [35,70] (Table 3). Therefore, further studies are needed to clarify the effects of cytokinin addition on the induction and development of embryogenic lines in *A. aculeata*.

Using picloram as a growth regulator was the most used and the best treatment for callus and somatic embryo induction in the different articles analyzed (Table 3 and Table 4), improving *A. aculeata* callus and somatic embryo formation in a concentration range from 9 μM to 450 μM [34,35,36,70,71,74]. Picloram is a systemic and selective herbicide that acts as an auxin-type growth regulator, inducing rapid cell proliferation and elongation, which may lead to callus tissue formation, and affecting other morphogenetic processes, with high efficacy due to its high mobility and resistance to degradation within the plant [76].

In 71.43% of the articles, somatic embryos were formed during callus multiplication, not requiring a specific induction medium for somatic embryo formation after callus multiplication. Some of these articles reported the asynchronous formation of somatic embryos, which can cause difficulty in obtaining a high number of regenerated plants [35,36]. Although the reviewed studies did not evaluate pre-treatment with osmotic agents or staged hormone withdrawal as synchronized induction strategies for somatic embryo formation, these approaches are documented in other palm species and could be promising for the macaw palm [77,78]. Therefore, exploring such strategies represents a potential avenue for future research aimed at overcoming the limitation of asynchronous embryo development in *A. aculeata*.

Adding putrescine with picloram promoted callus multiplication cycles, somatic embryo formation, and plant regeneration (Table 4), demonstrating its potential as a growth regulator for the asexual propagation of *A. aculeata* [34]. Putrescine is a polyamine, and polyamines are a type of plant growth regulator that help protect cells from stress, preventing DNA damage and regulating DNA methylation, contributing to somatic embryo formation and maturation [34,79]. On the other hand, Granja et al. [35] used putrescine during somatic embryo germination/regeneration and obtained a low rate of germinated somatic embryos, and the plantlets’ regeneration was defective. Given the role of polyamines in important physiological processes, more studies exploring their application in *A. aculeata* micropropagation should improve propagation protocols.

Plant growth regulators were not used in four experiments (57.14% of the analyzed articles) during somatic embryo germination and regeneration stage (Figure 10). Among these, 50% of the experiments failed to regenerate plants, 25% achieved only germination, and 25% regenerated plants with the development of roots and shoots. Among the experiments that used plant growth regulators during the somatic embryo germination and regeneration stage (28.57% of the analyzed articles), 50% germinated somatic embryos but presented defective plant regeneration, while 50% successfully regenerated plants with well-developed shoots and roots (Table 4). Only one of the analyzed articles (14.29%) did not assess the somatic embryos’ germination/regeneration.

Additionally, the type and developmental stage of the explant are equally important. Juvenile tissues, like zygotic embryos or immature leaves, often have a greater capacity for morphogenic development than do adult tissues. Furthermore, the effectiveness of both embryogenic induction and regeneration during culture can be impacted by environmental factors, such as temperature, photoperiod, and light quality. The conversion of embryos into viable plantlets and callus stability are also impacted by the frequency of subculturing and maintenance techniques.

Regarding the age of the mother plant of *A. aculeata*, the younger the mother plant, the higher the percentage of embryogenic calli and the lower the percentage of oxidized explants, specifically utilizing 2-year-old explants [34]. However, the reviewed literature does not explicitly detail whether this effect was quantified across multiple genotypes or how ‘older’ plants were biologically defined for comparative purposes within the summarized studies, indicating a potential area for more systematic investigation in future research. For context, a macaw palm plant is considered adult and productive when it reaches approximately five years of age, starting to bear fruit at this stage and potentially producing for many decades. The explant types used to induce *A. aculeata* callus were immature and unexpanded leaves (57.14% of the micropropagation articles) and zigotic embryos (42.86%) (Table 3). When using immature and unexpanded leaves, the explants can be obtained from different regions of the palm heart. Meira et al. [36] observed that the region distant from the meristem showed the greatest calli production response.

The temperature of 25 °C was predominantly used in all stages of *A. aculeata* micropropagation. Only one study evaluated 27 °C for all stages, while another investigated 30 °C during somatic embryo germination and regeneration (Table 3 and Table 4). It is impossible to affirm whether temperature affects *A. aculeata* micropropagation, as none of the analyzed articles investigated the effects of different temperatures. This deficiency of investigations on different temperatures for *A. aculeata* micropropagation highlights a knowledge gap to be explored in future studies.

Light conditions (including both dark and light conditions) varied mainly according to the micropropagation stage. Dark was predominantly used during callus induction and multiplication, while light or 16 h photoperiods were predominant during somatic embryo induction, maturation, and germination/regeneration (Table 3 and Table 4). Moura et al. [74] compared dark and a 16 h photoperiod for *A. aculeata* somatic embryo induction. They observed that in the dark, there was higher somatic embryo formation; however, these embryos exhibited a multicellular origin aspect and shared the protoderm with the mother tissue. In contrast, the 16 h photoperiod resulted in a lower somatic embryo formation, but these embryos had a unicellular origin aspect and did not share the protoderm with the mother tissue. These findings highlight the important role that light plays in somatic embryogenesis, affecting not only embryo formation but also its development and structural integrity. Proper light conditions can regulate hormonal balance, gene expression, and oxidative stress responses, enhancing somatic embryo formation and maturation, as well as their conversion into viable plants [80]. Most of the articles (71.43%) reported somatic embryo formation during the multiplication stage, which occurred predominantly in the dark (Table 4). In this context, experiments should be conducted to study *A. aculeata* callus multiplication under different light conditions to identify the best conditions for callus multiplication and somatic embryo formation.

Therefore, successful micropropagation of *A. aculeata* requires an integrated approach that considers the interplay between disinfection protocols, culture medium formulation, and growth regulator combinations, as well as additional factors such as explant source, developmental stage, and in vitro environmental conditions. This systems perspective not only improves coherence in experimental design but also highlights the main challenges to be overcome for the advancement of asexual propagation in this species.

## 3. Conclusions and Future Perspectives

The propagation of *A. aculeata* remains a major bottleneck for its domestication and commercial expansion. Sexual propagation is the most widely studied approach; however, its efficiency is strongly limited by non-deep physiological dormancy in the seeds of this palm. As it is still in the process of domestication, high heterogeneity in fruit production is observed, which greatly affects the different depths of dormancy observed in fruits from the same individual. The literature analyzed in this review indicates a clear methodological hierarchy for sexual propagation, in which strategies that directly eliminate the mechanical constraint imposed on embryo growth are consistently more effective than those acting indirectly through environmental or hormonal modulation. In this context, approaches targeting the operculum represent the most reliable pathway for improving germination performance, while temperature and growth regulators play secondary, modulatory roles once this primary constraint is removed.

Although less studied, asexual propagation is essential for the clonal multiplication of superior genotypes. Cutting propagation showed limited success due to low root emission, indicating a need for improvement, such as the potential use of plant growth regulators. On the other hand, somatic embryogenesis-based micropropagation showed promise as an asexual propagation method. High embryogenic potential was demonstrated by protocols based on picloram-induced callus formation from juvenile explants, primarily using Y3 medium and activated charcoal. The efficiency and reproducibility of these protocols are limited by some issues, such as asynchronous somatic embryo development, low conversion rates into viable plantlets, and frequent abnormalities.

Future studies should focus on: (a) integrating mechanical, hormonal, and environmental strategies to improve sexual propagation; (b) optimizing somatic embryogenesis micropropagation protocols, particularly the synchronization of embryos formation, as well as their maturation and regeneration; and (c) systematically evaluating the effects of culture media composition, growth regulators, and environmental conditions in macaw palm propagation. Overcoming these challenges will be crucial to enabling large-scale propagation and supporting the sustainable commercial use of *A. aculeata*.

## 4. Material and Methods

### 4.1. Data Source and Search Strategy

**Figure 11:**
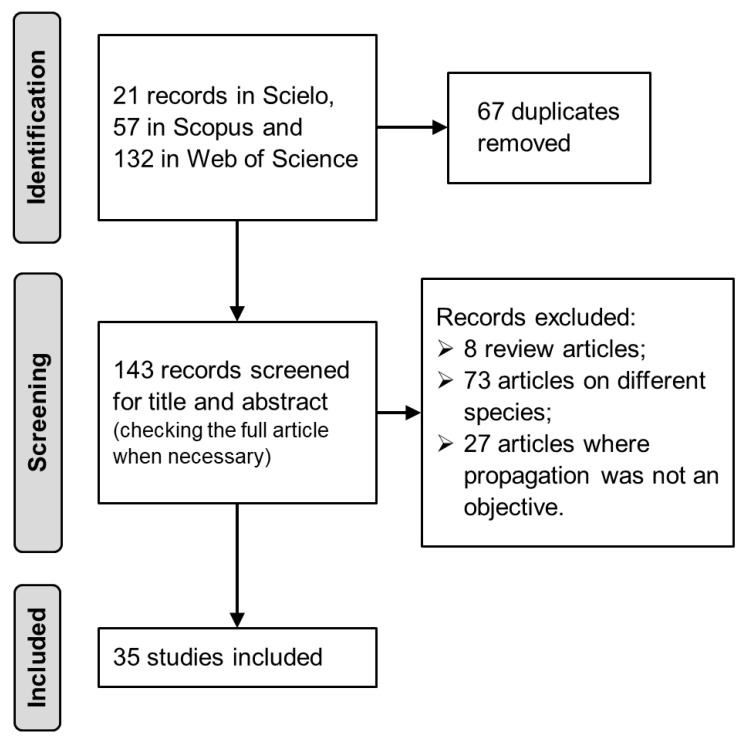
Flow diagram of the article selection process for this integrative review. The process involved identifying records from different databases, removing duplicates, screening based on titles and abstracts, and excluding studies that did not meet the eligibility criteria, resulting in the final selection of studies included in the review.

An integrative review, supported by bibliometrics, of *A. aculeata* propagation was performed. The primary research question that covers all the aspects of *A. aculeata* propagation is the following: “What are the most studied and effective methods and experimental conditions for *A. aculeata* sexual and asexual propagation?”. The articles selection was conducted based on the PICO strategy, dividing up the research question into (i) Population: *A. aculeata*; (ii) Intervention: propagation methods (sexual and asexual) and experimental conditions influencing propagation; (iii) Comparison: no intervention, using control treatments or environmental conditions; and (iv) Outcome: propagation data such as germination, plantlets emergence, sprouting, somatic embryogenesis. The search was conducted from January to February 2025. Searches were conducted in Scopus (titles, abstracts, and keywords), SciELO (all indexes), and Web of Science (all fields). No filters were applied during the database searches. The search criteria included a range of keywords related to the species *A. aculeata* and its vernacular names, as well as keywords related to propagation as follows: (“*Acrocomia aculeata*” OR “macaúba” OR “macaw palm” OR “bocaiuva” OR “macaiba”) AND (“propagation” OR “germination” OR “multiplication” OR “reproduction” OR “micropropagation” OR “tissue culture”). Two reviewers independently conducted the search, evaluation, and selection of articles on different dates. Only scientific articles that evaluated the sexual and/or asexual propagation of *A. aculeata* were selected, excluding review articles, studies focused on other species, and articles in which the propagation of *A. aculeata* was not the main objective. This article selection process was performed according to a defined set of criteria (Figure 11).

The information extracted from the articles reviewed in this study was organized in tables and verified by two independent evaluators using a double-entry method, allowing error detection and correction through a second check of the sources. The articles were categorized according to the studied variables to systematize and facilitate result interpretation. The following data were recorded: first author and year of publication, city and country where *A. aculeata* samples were collected, type of propagation method (sexual or asexual), number of experiments for each propagation method per article, whether the experiments were conducted in vitro or in vivo, experimental conditions and treatments (such as temperature, light conditions, substrate or culture medium, use of plant growth regulators, among other information), and results obtained.

### 4.2. Bibliometric Analysis

All bibliometric analyses were performed using the R software [81] (version 4.4.0; R Foundation for Statistical Computing, Vienna, Austria) within the RStudio environment [82] (version 2025.09.2; Posit Software, PBC, Boston, MA, USA), employing the bibliometrix package (version 5.2.1) for science mapping and network analysis [83]. A descriptive bibliometric analysis was conducted using the *biblioAnalysis()* function to obtain information on publication trends, sources, authorship patterns, collaboration metrics, and keyword occurrence. Summary statistics were generated using the *summary()* function.

### 4.3. Data Cleaning and Keyword Standardization

To improve the robustness of thematic and network analyses, generic and non-informative terms were removed from the keyword fields (Keywords Plus and Author Keywords). Terms related to species name (e.g., “*Acrocomia aculeata*”), common names (e.g., “macaw palm”), geographic descriptors at the country level (e.g., “Brazil”), and general descriptors (e.g., “palm tree”) were excluded using regular expressions. This cleaning step reduced thematic noise and enhanced the detection of meaningful conceptual patterns.

### 4.4. Thematic Map Analysis

The conceptual structure of the research field was explored through a thematic map, generated using keyword co-word analysis based on Keywords Plus. Themes were positioned according to their centrality (degree of interaction with other themes) and density (internal development), allowing classification into motor, basic, niche, and emerging or declining themes.

### 4.5. Classification of Propagation Strategies

Publications were classified into sexual and asexual propagation strategies based on keyword matching across titles, abstracts, and keyword fields. Predefined sets of terms related to “zygotic embryogenesis, germination, seed dormancy, emergence, nursery, endocarp, operculum, protrusion, scarification, mechanical, acid scarification, stratification, substrates, germitest and sexual propagation” were used to identify sexual propagation studies, while terms “cutting propagation, tissue culture, somatic embryos germination, multiplication, induction, maturation, regeneration, somatic embryo, somatic embryogenesis, micropropagation, bioreactor, callus, explant, embryogenesis, asexual propagation” were used to classify asexual studies.

### 4.6. Temporal Analysis of Propagation Strategies

To assess temporal trends, the annual number of publications was calculated separately for sexual and asexual propagation strategies. A line graph was constructed to illustrate changes in research emphasis over time.

### 4.7. Keyword Co-Occurrence Network Analysis

Keyword co-occurrence networks were constructed separately for sexual and asexual propagation studies using the *biblioNetwork()* function. Networks were visualized using a force-directed layout, with node size proportional to keyword frequency and edge thickness reflecting co-occurrence strength. Clusters were identified using the Walktrap algorithm, enabling comparison of thematic organization between propagation strategies.

## Figures and Tables

**Table 1:** Summary of best reported methods and results for ex vitro sexual propagation of *A. aculeata*.

Disinfestation	Dormancy Overcoming	Substrates Types	Temperature (°C)	Light Conditions	Duration Time	Main Results	Source
Sodium hypochlorite (5%)	Operculum removal	Vermiculite and iron ore tailings	25	Photoperiod of 12 h	56 days	Seed germination rates were 65% in iron ore tailings and 68% in vermiculite	[16]
Sodium hypochlorite (6%)	Operculum removal	Vermiculite	Germinators adjusted to 20, 25, 30, 35, or 40 °C. After 30 days, all treatments were transferred to 30 °C.	Not informed	60 days	For seeds without an operculum, germination at 60 days ranged from 70% to 90%, with no significant difference among treatments.	[45]
Sodium hypochlorite (6%)	Operculum removal	Vermiculite	30	Dark	21 days	±80% germination rate	[19]
Sodium hypochlorite (6%)	GA_3_ (2000 mg L^−1^)	Vermiculite	30	Dark	60 days	60% of germination	[19]
Sodium hypochlorite (5%)	Operculum removal + GA_3_ (0.5%)	Blotting paper	27	Not informed	29 days	80% of germination	[31]
Sodium hypochlorite (6%)	Stratification at 35 °C for 60 days	Vermiculite	30	Not informed	12 weeks	25% of germination	[44]
Sodium hypochlorite (5%)	Operculum removal + GA_3_ (0.5%)	Germitest^®^ paper	28	Dark	30 days	Best seed conservation occurred at 6–8% moisture, with germination declining from ±60% to 40% over 12 months at room temperature	[33]
Sodium hypochlorite (5%)	Operculum removal + GA_3_ (0.5%)	Germitest^®^ paper	28	Dark	30 days	82.66% of germination and 2.23 of GSI were obtained at 20 °C and 55% RH	[33]
Absolute ethyl alcohol + sodium hypochlorite (2.5%)	Operculum removal + GA_3_ (100 mg L^−1^)	Blotting paper	35	Not informed	28 days	13.75% of germination	[27]
Sodium hypochlorite (6%)	GA_3_ (2000 mg L^−1^)	Vermiculite	30	Photoperiod of 12 h	21 weeks	37% of germination	[42]
Sodium hypochlorite (6%)	Operculum removal	Vermiculite	30	Dark	15 days	86% of germination	[40]
Sodium hypochlorite (6%)	Operculum removal + soaking in water for 96 h	Vermiculite	30	Not informed	8 days	60% of germination	[41]
Sodium hypochlorite (6%)	GA_3_ (2000 mg L^−1^) for 24 h	Vermiculite	30	Not informed	60 days	20% of germination	[41]
Sodium hypochlorite (5%)	Operculum removal + GA_3_ (1000 mg L^−1^)	Vermiculite	30	Not informed	84 days	±59.07% of germination	[30]
Chlorine solution (6%)	Operculum removal + GA_3_ (0, 2000 or 5000 mg L^−1^)	Vermiculite	30	Not informed	18 weeks	Opercular tegument removal yielded the highest germination (35%), independent of GA_3_. In intact opercular teguments, GA_3_ increased germination from 15% (control, without GA_3_) to 25%.	[59]
Not informed	Soaking times for seeds in water (0, 2, 4, 6, 8 and 10 days)	Germitest^®^ paper	30	Not informed	Not informed	Germination and GSI decreased with increasing soaking time, from 10% and 0.06 (day 0) to 2.5% and 0.03 after 10 days of imbibition	[15]
Chlorine solution (6%)	Pyrenes pre-soaking in GA_3_ (≥100 mg L^−1^) for 24 h or soaking in water at 40 °C for 12 h	Clay soil + sand	25	Not informed	13 months	Proved to be simple and effective procedures for production of seedlings	[39]
Chlorine solution (6%)	Operculum removal + GA_3_ (2000 mg L^−1^) soaking for 24 h followed by a repeated GA_3_ application after 4 weeks	Vermiculite	30	Not informed	126 days	>50% of germination within eight weeks	[39]

GSI = germination speed index; RH = relative humidity.

**Table 2:** Summary of the best methods and results reported for the in vitro sexual propagation of *A. aculeata*.

Pre-Germination	Disinfection Protocol	Explant	Culture Medium	AC (g L^−1^)	PGR	T (°C)	Light Condition	Period	Main Results	Source
Without	Not informed	Embryos	MS 75% + organic substances	0	Without	30	Dark	30 days	100% of viable embryos (i.e., capable of elongation)	[45]
Without	Not informed	Embryos	MS 75%	3	Without	30	Dark	30 days	89% embryo viability and 80% of vigor	[45]
Embryos hydrated for 15 h (60% moisture) followed by 6 h desiccation (11% moisture) prior to cryopreservation	Seeds: Alcohol (70%) + Sodium hypochlorite (2.5%)	Embryos	Modified Y3	2.5	Without	25 ± 2	Dark for 30 days followed by 16 h photoperiod	>30 days	81% (1 h) and 75% (360 days) germination post-cryopreservation, with no significant difference	[65]
Heat treatment (temperature regimes: 35, 40, 20/30, 18/35 °C) or maintained at room temperature (±22 °C)	Embryos: Chlorine solution (0.5%)	Embryos	MS 100%	3	Without	30	Dark	30 days	The initial embryo elongation rate (control) was 95.8%. Embryo elongation over time at 25, 20/30, and 18/35 °C was similar to the control, but decreased at 35 °C after 60 days and at 40 °C after 15 days	[44]
Without	Seeds: Alcohol (90%) + Sodium hypochlorite (20%)	Embryos	MS 100%	2	BAP (1 pmm)	25	Dark for 20 days followed by 16 h photoperiod	40 days	5 to 6 cm of root growth	[53]
Different storage conditions (fruits stored in the shade at room temperature (27 ± 2 °C) or in a cold chamber (12–15 °C) in the dark) for 30 days	Embryos: Sodium hypochlorite (1%) with Tween 20^®^	Embryos	MS 100% with different concentration of sucrose (5, 10, 15, 20, 25 and 30 g L^−1^)	2	Without	27	Dark for 30 days followed by 16 h photoperiod	90 days	84% of germination with room-temperature fruit storage. Highest germination (82%) and embryo-to-seedling conversion (42%) at 30 g L^−1^ sucrose.	[67]
Without	Seeds: Sodium hypochlorite (6%) Embryos: Ascorbic acid solution (100 mg L^−1^) + Chlorine solution (0.5%)	Embryos (control), embryo + tegument, and embryo + endosperm	MS 75%	6	Without	30	Dark	30 days	Embryos cultivation in contact with seed structures appeared to have no effect on their germination or development	[32]
		Embryos	MS 75%	6	GA_3_ (0 mg L^−1^)	30	Dark for 30 days followed by 12 h photoperiod	30 days	87% of the embryos showed cotyledonary petiole elongation	[32]
Stored in a cold-chamber at 10 °C for one full year	Seeds: Chlorine solution (6%)	Embryos	MS 75%	3	Without	30	Dark	30 days	85% of embryo viability	[58]
Without	Seeds: Sodium hypochlorite (1%) Embryos: Sodium hypochlorite (0.5%) with Tween 20^®^	Embryos	WPM 100%	1	Biobras 16^®^ (0.5 mg L^−1^)	25	Dark for 30 days followed by 16 h photoperiod	90 days	80% of germination and 53.1% of normal seedlings	[63]
Without	Embryos: Commercial sodium hypochlorite (20%)	Embryos	MS (50 and 100%) + Coconut water (0, 50, 100 and 150 mL L^−1^)	3	Without	25	16 h photoperiod	90 days	Highest germination (95.6%) after 60 days regardless of medium composition; highest proportion of normal seedlings (83%) obtained with 50% MS + 50 mL L^−1^ coconut water.	[56]
Without	Embryos: Ascorbic acid solution (100 ppm) + Chlorine solution (0.25%)	Embryos	MS 75%	3	Without	30	Dark	30 days	Germination ranged from 38–68% in embryos with 7.9% water content to 82–96% in embryos with 20.4% water content	[29]

AC = activated charcoal; PGR = plant growth regulator; T = temperature.

**Table 3:** Summary of the best methods and results reported for the micropropagation stage of *A. aculeata* callus induction.

Explant Type	Disinfection Protocol	Culture Medium	Activated Charcoal	PGR	Temperature (°C)	Light Conditions	Duration Time	Main Results	Source
Immature and unexpanded leaves (2-year-old explants)	Sodium hypochlorite (1%)	Y3	0 g	Picloram (18 and 36 μM)	27 ± 1	Dark	90 days	40% callus production	[34]
Immature and unexpanded leaves	Alcohol (70%) + Sodium hypochlorite (1.5%)	modified Y3 with Fe-EDTA + vitamins of MS	2.5 g L^−1^	Picloram (450 μM)	25 ± 2	Dark	9 months	64.9% callus production	[36]
Immature and unexpanded leaves	Alcohol (70%) + Sodium hypochlorite (2.5%)	Y3 + vitamins of MS	2.5 g L^−1^	Picloram (450 μM)	25 ± 2	Dark	180 days	There was callus formation, but the percentage was not reported	[71]
Zygotic embryos	Seeds: Commercial detergent + Sodium hypochlorite (2.5%) Zygotic embryo: Sodium hypochlorite (0.1%)	Y3	0 g	Picloram (9 μM) + 2iP (0.9 μM)	25 ± 2	Dark	90 days	46% callus production	[35]
Immature and unexpanded leaves (12-week old seedlings)	Seeds: Alcohol (70%) + Sodium hypochlorite (6%) with 0.1% Tween 20^®^	Y3	1.5 g L^−1^	Picloram (150 and 300 µM)	Not informed	Dark	12 weeks	>55% callus production	[70]
Zygotic embryos	Seeds: Detertec (50%) + Mercuric chloride (0.01%) + Sodium hypochlorite (6%)	Y3	0 g	2,4-D (9 μM) + TDZ (1 mM)	25 ± 2	Dark	60 days	61.9% callus production	[73]
		Y3	0 g	Picloram (9 μM)	25 ± 2	Dark	60 days	47% callus production	
Zygotic embryos		Y3	0 g	Picloram (9 μM)	25 ± 2	Dark	60 days	75 ± 4.5% callus production	[74]

PGR = plant growth regulators; Picloram = 4-amino-3,5,6-trichloropicolonic acid; 2,4-D = 2,4-dichlorophenoxyacetic acid; 2iP = 2-isopenteniladenina; TDZ = Thidiazuron.

**Table 4:** Summary of the best methods and results reported for each stage of *A. aculeata* micropropagation, except callus induction, which is presented in Table 3.

Microprop. Stage	Culture Medium	Activated Charcoal	PGR	Temperature (°C)	Light Conditions	Duration Time	Main Results	Source
Callus multiplication	Y3	0 g	Picloram (18 μM) + putrescine (1 mM)	27 ± 1	Dark	180 days	93.75% somatic embryo formation	[34]
	modified Y3 with Fe-EDTA and vitamins of MS	2.5 g L^−1^	Picloram (450 μM)	25 ± 2	Dark	4 months	Increment of 5.81 times the initial calli mass; Asynchronic formation of somatic embryos	[36]
	Y3 with vitamins of MS	2.5 g L^−1^	Picloram (450 μM)	25 ± 2	Dark	Not informed	Formation of somatic embryos	[71]
	MS	0 g	Picloram (18 μM)	25 ± 2	Dark	45 days	Asynchronic formation of somatic embryos	[35]
	Y3	0.3 g L^−1^	Picloram (75 μM)	Not informed	Light	8 weeks	Formation of nodular calli with embryogenic clusters	[70]
Somatic embryo induction	Y3	3.0 g L^−1^	Picloram (9 μM)	25 ± 2	16 h photoperiod	120 days	9% somatic embryo production	[73]
	Y3	0 g	Picloram (9 μM)	25 ± 2	Dark	120 days	59.9 ± 10% somatic embryo formation with multicellular origin aspect, but shared the protoderm with the mother tissue	[74]
	Y3	3.0 g L^−1^	Picloram (9 μM)	25 ± 2	16 h photoperiod	120 days	9 ± 3.5% somatic embryo formation with unicellular origin aspect	[74]
Somatic embryo maturation	MS	3.0 g L^−1^	2iP (2.5 μM) + NAA (5.5 μM) + putrescine (1000 μM)	25 ± 2	16 h photoperiod	60 days	28.08% of regenerated somatic embryos and 4.665% of pre-germinated embryo	[35]
	Y3	0 g	Picloram (75 μM) + 2iP (12.5 μM)	Not informed	Light	4 weeks	24% of the callus produced somatic embryos, with an average of 38 somatic embryos per nodular callus	[70]
Somatic embryo germination/regeneration	Y3	3.0 g L^−1^	2,4-D (0.1 µM) + putrescine (1 mM)	27 ± 1	Dark for 60 days and, then, 16 h photoperiod	60 days	100% of somatic embryos were converted into plantlets with well-developed shoots and roots	[34]
	Y3	2.5 g L^−1^	Without PGR	25 ± 2	Ligh	>1 month	Germinating somatic embryos went into senescence and died after a few weeks of germination	[36]
	MS	0 g	2iP (0.25 μM) + GA_3_ (0.55 μM) + putrescine (1000 μM)	30 ± 2	16 h photoperiod	60 days	14.938% of germinated somatic embryos (after 30 days); Plantlets’ regeneration was defective (after 60 days)	[35]
	Y3	1.0 g L^−1^	Without PGR	25 ± 2	Light	8 weeks	18% of somatic embryos converted into plants but did not survive acclimation	[70]
	Y3	3.0 g L^−1^	Without PGR	25 ± 2	16 h photoperiod	30 days	49.3% of the somatic embryos started germination, but only few embryos completed the germination; Plantlets appeared to be normal with growing roots and shoots	[73]
	Y3	3.0 g L^−1^	Without PGR	25 ± 2	16 h photoperiod	15 days	50 ± 5% of the somatic embryo with unicellular origin aspect germinated	[74]

Microprop. = micropropagation; PGR = plant growth regulators; Picloram = 4-amino-3,5,6-trichloropicolonic acid; 2,4-D = 2,4-dichlorophenoxyacetic acid; BAP = 6-Benzylaminopurine; 2iP = 2-isopenteniladenina; GA_3_ = Gibberellic acid.

## Data Availability

Data will be made available on request.

## Supplementary Materials

The following supporting information can be downloaded at: https://www.mdpi.com/article/10.3390/plants15030488/s1, Figure S1: Keyword co-occurrence networks by propagation strategy in *A. aculeata*.; Figure S2: Number of publications by institution in studies on *A. aculeata* propagation.
