# Supplementary material for: Macaw Palm Propagation Strategies: Advances, Gaps, and Future Directions for a Promising Oleaginous Crop—A Review
Source: Plants (Basel). 2026 Feb 5;15(3):488. doi: 10.3390/plants15030488 (PMC12899517; doi:10.3390/plants15030488)
Supplement: Supplementary file 1 [file plants-15-00488-s001.zip › plants-4100689-supplementary.pdf]

Supplementary materials

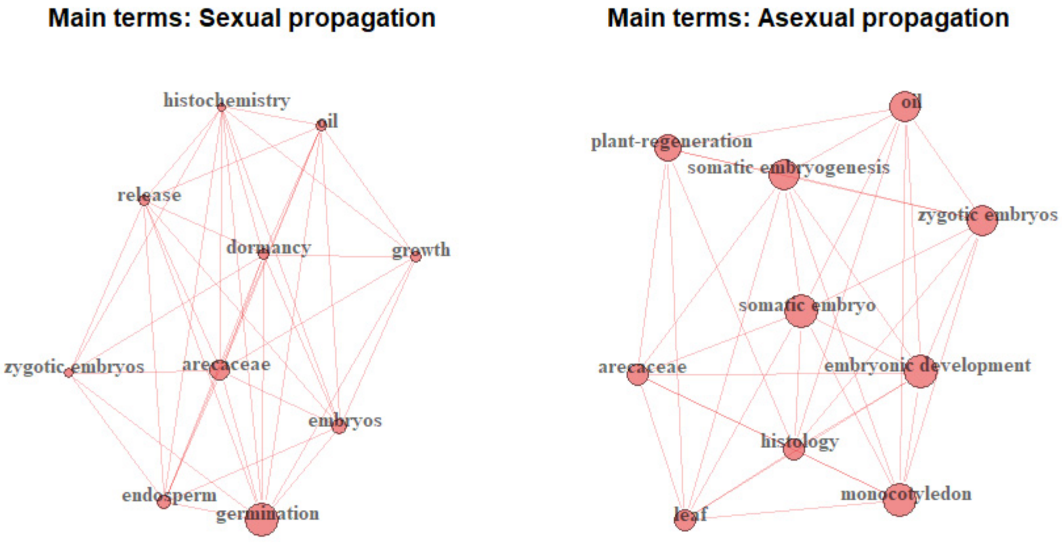

Figure S1. Keyword co-occurrence networks by propagation strategy in *A. aculeata*.

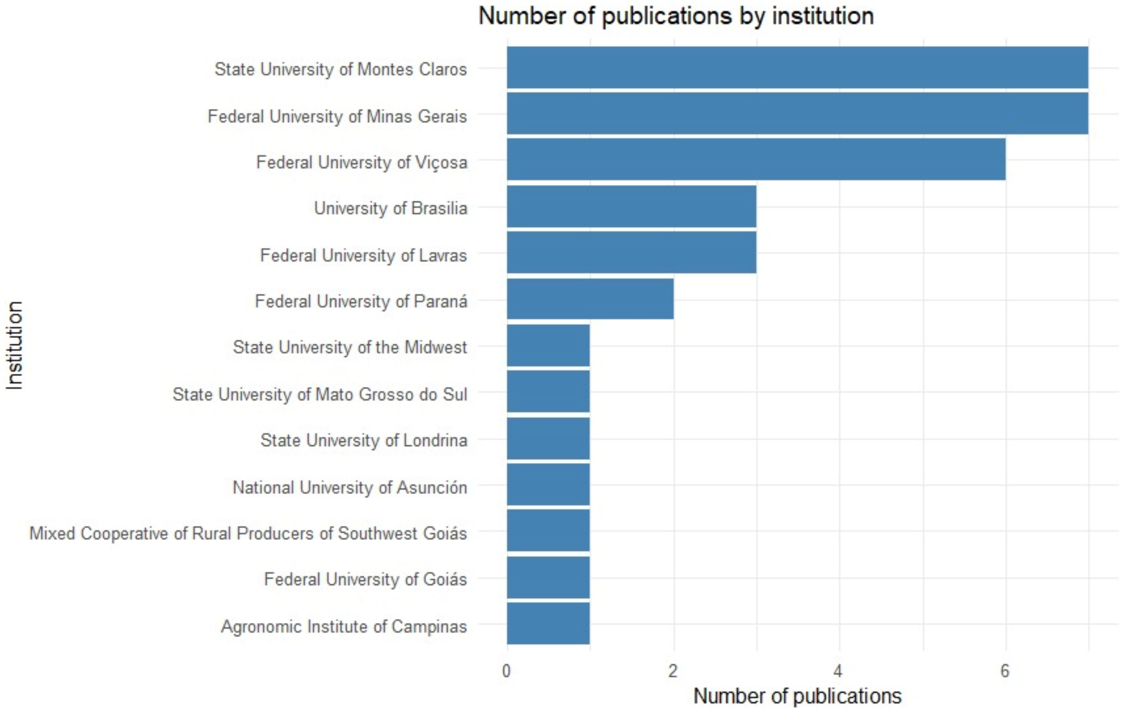

Figure S2. Number of publications by institution in studies on *A. aculeata* propagation.
